# In vitro comparison of fixation techniques for stabilization of Le Fort I osteotomy in short and large advancements of the maxilla

**DOI:** 10.1007/s10006-026-01596-8

**Published:** 2026-07-17

**Authors:** Misha L. Tan, Max P.A. Balkenende, Damir Delic, Karel Kuik, Cornelis J. Kleverlaan, Jan de Lange, Jean-Pierre T.F. Ho

**Affiliations:** 1 Department of Oral and Maxillofacial Surgery, Amsterdam UMC, location AMC, Meibergdreef 9, Amsterdam, 1105 AZ the Netherlands; 2 Department of Dental Materials, Faculty of Dentistry, Academic Center for Dentistry Amsterdam (ACTA), Gustav Mahlerlaan 3004, Amsterdam, 1081 LA The Netherlands; 3 Department of Oral and Maxillofacial Surgery, Noordwest Ziekenhuisgroep, Wilhelminalaan 12 1815JD, Alkmaar, The Netherlands

**Keywords:** Maxilla, Le Fort I, Osteotomy, Biomechanical evaluation, Fixation techniques, Rigidity

## Abstract

**Purpose:**

Le Fort I osteotomy is widely performed in orthognathic surgery for maxillary repositioning and for the treatment of dentofacial deformities and obstructive sleep apnea (OSA). Postoperative relapse remains a concern, particularly after large maxillary advancements, highlighting the importance of rigid fixation. This study aimed to determine which fixation technique provides the greatest rigidity.

**Methods:**

In this in vitro study, the biomechanical rigidity of five commonly used fixation techniques for Le Fort I osteotomies was evaluated using 150 polyurethane specimens with advancements of 5 mm and 10 mm. The techniques included two L-plates (A), four L-plates of different thicknesses (B and C), two precontoured Le Fort I plates (D), and a hybrid configuration with two precontoured Le Fort I plates combined with two L-plates (E). Resistance to vertical displacement was measured at 1, 3, and 5 mm using a biomechanical testing machine.

**Results:**

Fixation E consistently showed the highest resistance across both advancement distances and all displacement intervals, significantly outperforming fixations A, C, and D. Fixation B demonstrated resistance comparable to fixation E, whereas fixation C showed inferior performance despite a similar configuration. Fixations A and D showed the lowest resistance, particularly at 10 mm advancement.

**Conclusion:**

Overall, fixation E provided the greatest rigidity, while techniques relying on only two plates were biomechanically less effective. Further clinical studies are required to determine the clinical relevance of these findings.

## Introduction

Le Fort I osteotomy is the most commonly performed procedure in orthognathic surgery used to alter the position of the maxilla [1, 2]. It is often combined with a bilateral sagittal split osteotomy (BSSO) of the mandible and/or genioplasty to correct complex dentofacial deformities, aiming to restore both functional occlusion and facial aesthetics [2]. Additionally, a combination of a Le Fort I osteotomy and BSSO with substantial anterior advancement and not seldomly counterclockwise rotation – also known as a maxillomandibular advancement or MMA – is an established treatment modality for patients with obstructive sleep apnea (OSA) to increase upper airway [3–5].

Despite its widespread use, skeletal relapse following Le Fort I osteotomy remains a significant clinical challenge, with reported relapse rates ranging from 10% to 50% [6–8]. Larger maxillary advancements are particularly vulnerable to postoperative instability, with the majority of relapse occurring within the first year after surgery [7]. Insufficient rigidity at the osteosynthesis site not only contributes to relapse but also increases the risk of malunion or nonunion of the maxilla [9–11]. These complications can negatively affect occlusion, facial aesthetics, and functional outcomes, and, in the context of maxillomandibular advancement (MMA) procedures, may compromise the efficacy of obstructive sleep apnea treatment [4, 12]. Consequently, achieving adequate biomechanical rigidity is essential. Stable fixation promotes predictable bone healing, reduces the need for secondary interventions, and ensures long-term skeletal and functional stability [13, 14].

In current clinical practice, fixation techniques vary considerably between institutions and surgeons, often influenced by individual experience and training background [15]. Additionally, there is currently no consensus regarding the most rigid fixation technique [1, 16]. Some studies find that the absence of standardized guidelines leads to inconsistent fixation techniques, which probably contribute to variable postoperative stability outcomes [15]. Addressing this variability is needed to improve predictability and reduce complications [14, 15].

Biomechanical evaluation with polyurethane skulls offers a controlled and reproducible method to assess the mechanical performance and rigidity of different fixation techniques independently of patient-specific variables such as bone quality, healing capacity, and postoperative behaviour [17]. By isolating these mechanical factors, biomechanical studies can provide objective data on fixation strength, resistance and rigidity, which are critical to stability and reducing complications such as skeletal relapse and inadequate bone healing [15, 16].

This study aims to evaluate the biomechanical rigidity of five commonly used fixation techniques for maxillary stabilization following Le Fort I osteotomy in both small and large advancements. By identifying fixation techniques that provide optimal rigidity and resistance, this research seeks to support the development of evidence-based fixation protocols for improved surgical outcomes of the Le Fort I osteotomy.

## Methods

**Fig. 1:**
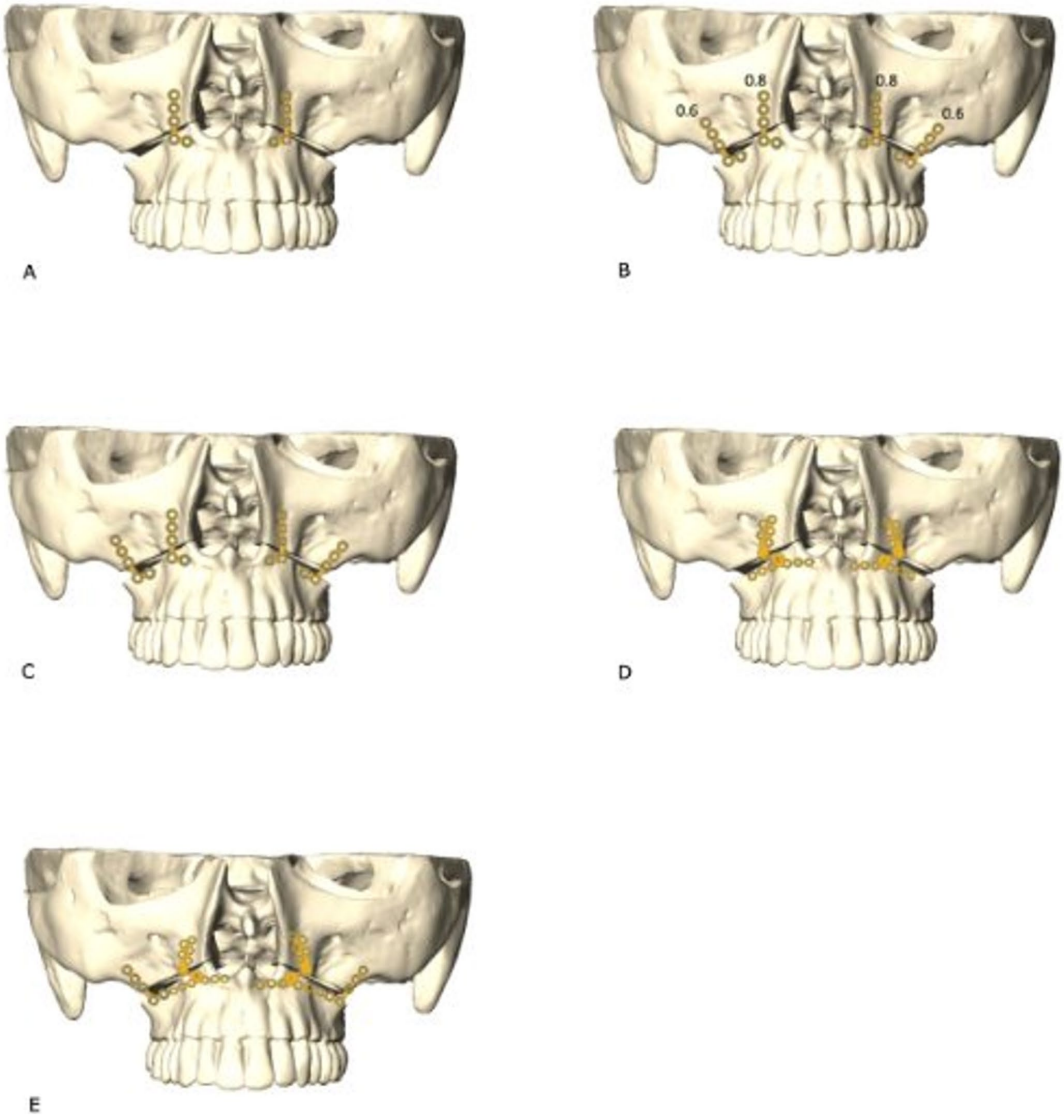
Schematic representation of five fixations techniques with standard off-the-shelf plates: **A** two L-plates (profile height of 0.8-mm); **B** four L-plates (profile height of 0.6-mm (outer plates) and 0.8-mm (inner plates)); **C**: four L-plates (profile height of 0.8-mm); **D**: two precontoured Le Fort I plates (profile height of 0.9-mm); **E**: two precontoured Le Fort I plates (profile height of 0.9-mm) combined with two L-plates (profile height of 0.8-mm)

In this in vitro study, 150 identical polyurethane models consisting of the cranium and maxilla were used for loading tests. A standardized Le Fort I osteotomy was performed by the manufacturer (Nacional Ossos, Jaù, Sao Paulo, Brazil). The models were divided among five fixation groups with 15 models each. A specific configuration of titanium plates was used for each group: (A) two L-plates (profile height of 0.8 mm); (B) four L-plates (profile height of 0.6 mm (outer plates) and 0.8 mm (inner plates)); (C) four L-plates (profile height of 0.8 mm); (D) two precontoured Le Fort I plates (profile height of 0.9 mm); (E) two precontoured Le Fort I plates (profile height of 0.9 mm) combined with two L-plates (profile height of 0.8 mm) (Fig. 1). Non-locking screws (1.7 mm) were used in all fixation techniques. All fixation materials were manufactured by Stryker Leibinger GmbH & Co. KG (Freiburg, Germany). All configurations were tested at two different advancements: short advancement (5 mm) and large advancement (10 mm).

**Fig. 2:**
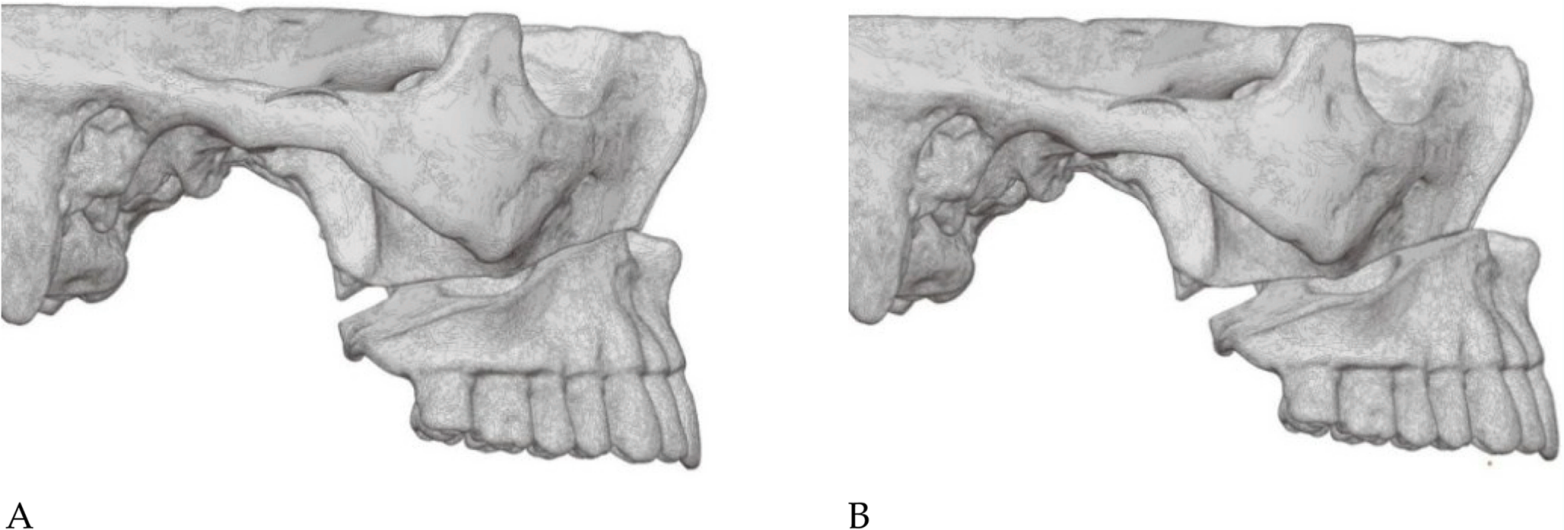
3D planned advancement of the maxilla with a counterclockwise. (**A**) 5-mm advancement. (**B**) 10-mm advancement

Both advancements were planned three-dimensionally (3D) using Blender software (Blender Foundation, version 4.2, Amsterdam, The Netherlands) (Fig. 2). Based on these virtual treatment plans, two models representing clinically relevant maxillary advancements were created. In the first model, the maxilla was advanced by 5 mm. To maintain paranasal bony contact, a 1-mm impaction was incorporated at the incisal point, followed by an 11° counterclockwise (CCW) rotation around the incisal point. This movement resulted in approximately 4 mm of inferior displacement at the mesiobuccal cusp of the first molars.

In the second model, the maxilla was advanced by 10 mm. To preserve paranasal bony contact, a 1.5-mm impaction was applied at the incisal point, followed by the same 11° CCW rotation around the incisal point. This resulted in approximately 3.5 mm of inferior displacement at the mesiobuccal cusp of the first molars.

**Fig. 3:**
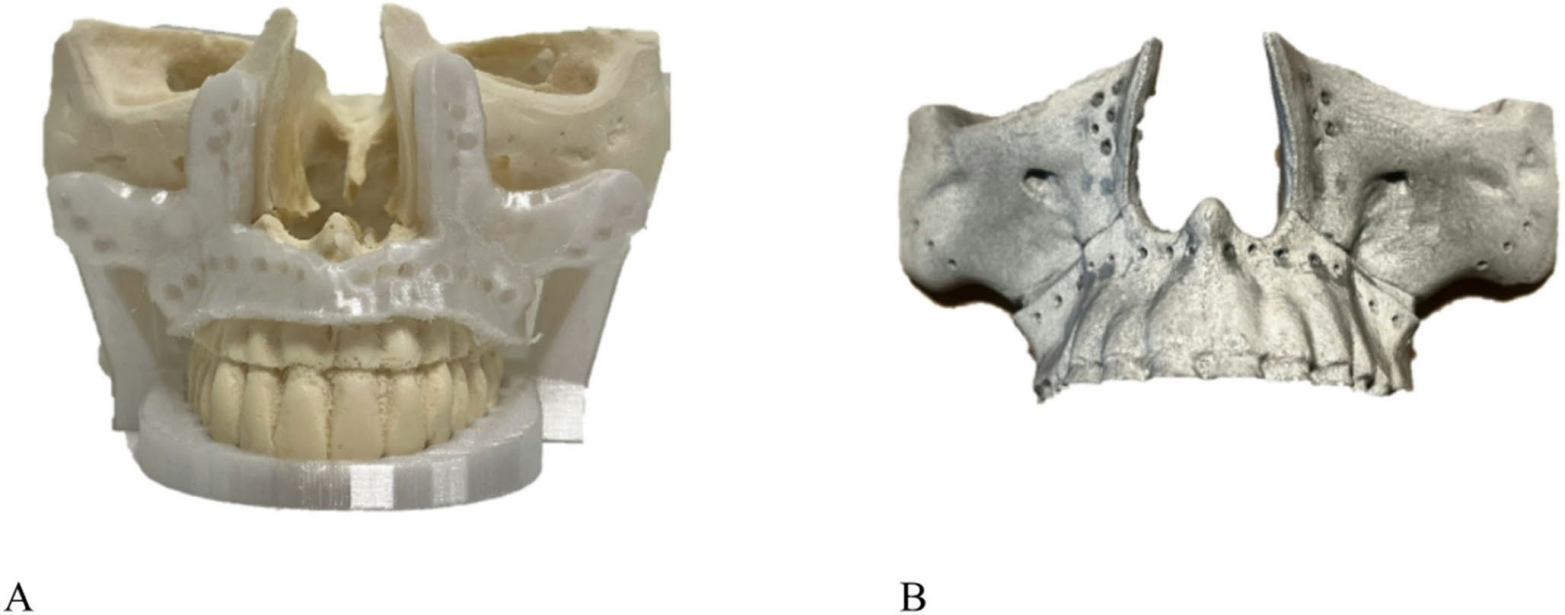
(**A**) A polyurethane model in the 3D printed drill jig. (**B**) A 3D printed bending template that includes a maxillary advancement and the screw positions

The 3D models for both advancement lengths were printed, and the positioning of screws and plates was determined by an experienced oral and maxillofacial surgeon (JH). To standardize drilling and plate bending, drill jigs and bending templates were designed with Blender software (version 4.2, Amsterdam, The Netherlands) and 3D-printed (Bambulab P1S, Shenzhen, China) in polylactic acid for each fixation technique (Fig. 3).

**Fig. 4:**
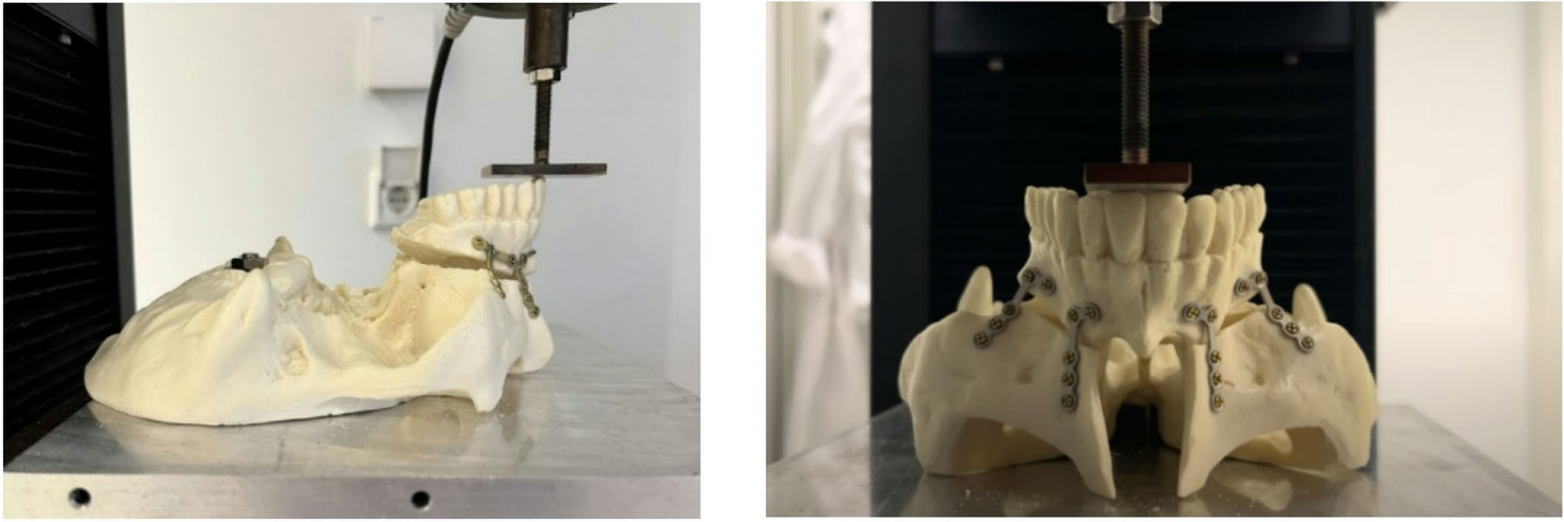
Experimental setup with fixation of the cranial segment. Vertical compressive forces are applied to the upper central incisors

Biomechanical testing was carried out using a biomechanical testing machine (Instron 6022, High Wycombe, UK). The cranial part of each skull model was immobilized, and vertical loading, at a speed of 1 mm/min, was applied to the upper central incisors (Fig. 4). Resistance forces, measured in Newtons, were recorded at 1-, 3-, and 5-mm displacements for all fixation techniques within both advancement groups. After testing, the skull models, plates, and screws were macroscopically examined for fracture or loosening.

Prior to initiation of the study, a power analysis was conducted using G*Power (version 3.1, Düsseldorf, Germany) to determine the minimum required sample size per advancement group. Based on a one-way ANOVA with an effect size of 0.40, a power of 0.80, an alpha level (α) of 0.05, and five groups, an effect size of 0.40 was deemed appropriate according to previous literature [18]. The analysis indicated that a minimum of 75 specimens per advancement group was required, corresponding to 15 skulls per fixation technique. Consequently, a total of 150 polyurethane skull models were included in this study.

Quantitative data were analyzed using the Statistical Package for the Social Sciences (SPSS, version 27, IBM, Chicago, IL, USA). The Kolmogorov–Smirnov test was employed to assess the normality of data distribution within each subgroup. For normally distributed data, means and standard deviations were calculated and analyzed via one-way ANOVA. Post hoc comparisons were performed using Tukey’s test at a 95% confidence interval (α = 0.05) to compare the five fixation techniques within each advancement group. For non-normally distributed data, the Kruskal–Wallis test was applied, followed by Dunn’s post hoc test. To compare differences between short and large advancements for each fixation technique, the Mann–Whitney U test was conducted. Since five separate Mann–Whitney U tests were performed (one per fixation), the Bonferroni correction was applied to control for type I error due to multiple comparisons. The adjusted significance level was set at α = 0.01 (0.05/5).

## Results

Mean forces in Newtons were measured at 1-mm, 3-mm, and 5-mm displacements for both short (5-mm) and large (10-mm) advancements (Table 1A-C). Fixation E showed the highest resistance forces at all displacements, significantly greater than Fixations A and D at all levels, and greater than Fixation C at 3-mm and 5-mm for both advancement lengths. No significant differences were found between Fixations B and E. Fixations A and D exhibited the lowest resistance forces consistently.

**Table 1:** Mean resistance using vertical loading measured in Newton. (A) 1 mm of displacement. (B) 3 mm of displacement. (C) 5 mm of displacement

A: 1 mm	5 mm advancement			10 mm advancement			
	Mean (SD)	Median (IQR)	*p*-value*	Mean (SD)	Median (IQR)	*p*-value*	*p*-value**
A (two L-plates)	17.6 (5.0)	17.0 (9.0)	B, C, E	12.1 (2.8)	12.9 (3.9)	B, C, E	0.004
B (four L-plates 0.6/0.8)	49.4 (9.18)	50.0 (15.3)	A, D	46.1 (13.1)	44.1 (17.9)	A, D	NS
C (four L-plates)	45.0 (6.36)	44.0 (6.0)	A, D	35.8 (7.42)	37.5 (11.3)	A, D	0.002
D (two Le Fort I plates)	25.9 (6.26)	28.6 (7.6)	B, C, E	12.9 (3.7)	12.5 (5.7)	B, C, E	< 0.001
E (two Le Fort + two L-plates)	71.6 (16.2)	71.7 (16.0)	A, D	58.0 (15.1)	58.7 (14.0)	A, D	NS
B: 3 mm	5 mm advancement			10 mm advancement			
	Mean (SD)	Median (IQR)	*p*-value*	Mean (SD)	Median (IQR)	*p*-value*	*p*-value**
A (two L-plates)	20.3 (3.7)	20.5 (5.8)	B, C, E	23.87 (3.6)	24.8 (5.1)	B, C, E	NS
B (four L-plates 0.6/0.8)	139.7 (24.3)	142.0 (40.5)	A, D	129.8 (27.0)	124.0 (41.5)	A, D	NS
C (four L-plates)	123.9 (21.9)	128.0 (27.0)	A, E	93.2 (15.0)	90.2 (22.0)	A, D, E	< 0.001
D (two Le Fort I plates)	48.5 (9.77)	51.6 (15.0)	B, E	23.2 (5.39)	23.8 (4.2)	B, C, E	< 0.001
E (two Le Fort + two L-plates)	218.3 (42.1)	208.7 (58.0)	A, C, D	163.9 (46.9)	151.6 (36.0)	A, C, D	< 0.001
C: 5 mm	5-mm advancement			10-mm advancement			
	Mean (SD)	Median (IQR)	*p*-value*	Mean (SD)	Median (IQR)	*p*-value*	*p*-value**
A (two L-plates)	17.2 (2.6)	17.0 (4.8)	B, C, E	24.4 (3.0)	24.5 (4.9)	B, C, E	< 0.001
B (four L-plates 0.6/0.8)	192.3 (33.9)	192.5 (40.3)	A, D	190.3 (36.6)	187.7 (53.0)	A, D	NS
C (four L-plates)	168.9 (30.3)	154.1 (38.4)	A, E	136.4 (18.6)	136.0 (30.0)	A, D, E	0.001
D (two Le Fort I plates)	49.6 (10.8)	48.0 (17.0)	B, E	24.3 (4.3)	24.9 (4.6)	B, C, E	< 0.001
E (two Le Fort + two L-plates)	306.4 (42.8)	297.0 (66.8)	A, C, D	219.3 (36.9)	211.5 (39.0)	A, C, D	< 0.001

SD standard deviation, IQR interquartile range

* Significant difference between fixation techniques. Statistical analysis performed using the Kruskal–Wallis test, followed by Dunn’s post hoc test for pairwise comparison

** Significant difference between 5.0-mm and 10.0-mm advancements determined using the Mann–Whitney U test

Comparing advancements, significantly lower forces were required for the large (10-mm) advancement versus the short (5-mm) advancement for Fixations C and D at all displacements; Fixation E at 3-mm and 5-mm; and Fixation A at 1-mm and 5-mm. Fixation B was the only fixation without significant force differences across advancements, indicating greater rigidity with increasing displacement.

A precise macroscopic evaluation post-loading revealed no fractures or screw loosening in the models or fixation plates.

## Discussion

This study aimed to evaluate the biomechanical rigidity of five different fixation techniques following Le Fort I osteotomy, comparing their performance under both small (5-mm) and large (10-mm) maxillary advancements. Fixation E demonstrated the highest biomechanical rigidity, followed by the four-plate configurations, while two-plate constructs showed inferior resistance, particularly in larger advancements.

These findings align with previous biomechanical and clinical studies emphasizing that increasing the number of fixation points enhances maxillary rigidity and postoperative stability [1, 19, 20]. Beyler et al. [1] reported improved postoperative stability following Le Fort I osteotomy with four-plate fixation compared with two-plate fixation. Ragaey and Van Sickels [21] demonstrated favourable stability in large maxillary advancements using a hybrid fixation approach consisting of precontoured and conventional plates. The superior performance of Fixation E in the present study is consistent with these observations and suggests that combining different plate designs may optimize force distribution and resistance to displacement.

Several finite element and biomechanical studies have further investigated the stability of Le Fort I fixation systems using different plate configurations, loading conditions, and advancement magnitudes [14, 16, 22–25]. Huang et al. [16] demonstrated that both fixation design and advancement magnitude influence displacement patterns and stress distribution, with four-plate configurations generally providing greater stability than less extensive fixation systems. Li et al. [14] showed that plate design significantly affects biomechanical performance by comparing conventional fixation systems with customized three-dimensional printed plates. Erguven et al. [25] used dynamic finite element analysis to simulate masticatory loading following Le Fort I advancement and reported increasing displacement and stress values with larger advancements, particularly within posterior fixation plates.

Collectively, these studies have provided valuable insight into stress distribution patterns, displacement behaviour, and the biomechanical consequences of maxillary advancement. However, most available evidence is derived from finite element analysis, which predicts biomechanical behaviour based on predefined material properties, loading conditions, and boundary constraints. While FEA enables highly standardized simulations and detailed analysis of stress concentrations within plates, screws, and surrounding bone [14, 16, 25], biomechanical testing provides direct physical measurements of construct rigidity and resistance to displacement under applied loading conditions [22–24]. Consequently, these methodologies should be regarded as complementary rather than competing approaches.

This study is the first providing a standardized experimental comparison of five clinically used fixation configurations, including conventional four-plate constructs, precontoured Le Fort I plates, and hybrid fixation systems, under both short and large advancements. Unlike previous FEA investigations, which primarily evaluated stress distribution and theoretical displacement patterns, our biomechanical setup directly quantified the force required to induce displacement of the osteotomized maxillary segment. In addition, all fixation strategies were assessed within a single experimental framework, allowing direct comparison of their relative rigidity under identical testing conditions. The findings thus provide direct experimental data that support and extend previous computational and clinical observations while offering clinically relevant information regarding the biomechanical performance of contemporary fixation techniques.

The clinical relevance of these findings need critical appraisal. First, the magnitude of forces applied to the maxilla, particularly at the region of the incisors, informs the required strength of fixation techniques. Previous studies [26, 27] report bite forces of approximately 200 N at the incisors under maximal voluntary clenching. For comparison, typical incisal forces during swallowing have been reported to be significantly lower, around 0.3 N, while routine incisal biting forces during functional activities range from 20 to 30 N [28]. Our data show that Fixation B requires around 190 N to cause a 5-mm displacement at both short and large advancements. In contrast, Fixation E displaced by only 1–3 mm under 200 N bite forces, especially in the larger advancement scenario. Postoperatively, patients are advised to follow a soft diet for six weeks, which helps reduce occlusal forces on the maxilla during early bone healing, thereby minimizing the risk of relapse and non-union. However, since complete bone union may take over 12 months [7], rigid fixation remains essential to maintain skeletal stability throughout this extended healing period.

Nevertheless, the level of rigidity is necessary in clinical practice remains debatable. While in vitro data allow comparison of mechanical performance, they do not directly indicate whether a given system would fail under physiological loading conditions. It is possible that all tested fixation techniques may provide sufficient stability in vivo, and that the actual threshold for osteosynthesis failure lies well above the forces typically encountered postoperatively.

While in this study, we chose an in vitro study design with polyurethane skull models to ensure reproducibility and standardisation. The use of polyurethane models for biomechanical evaluations is well documented in the literature [22, 23, 29, 30]. To further enhance consistency, custom drilling and bending guides were fabricated to ensure that all plates were positioned and shaped uniformly. While advantageous for biomechanical testing, polyurethane models have limitations [17]. The maxilla is subject to complex forces from multiple directions, including bite forces on the molars and dynamic influences from surrounding muscles, such as the masticatory muscles and tongue. Although posterior bite forces are generally higher than incisal forces, loading was applied at the incisors because this location creates a larger bending moment on the fixation construct due to its greater distance from the fixation sites and provides a standardized testing condition for comparing fixation techniques. Our experimental setup applied a single, unidirectional force in a smooth motion and did not simulate repeated or cyclic loading, which may contribute to plate fatigue over time [24, 25]. Long-term fatigue loading, which is critical for understanding clinical relapse, was also not evaluated.

Furthermore, patient-specific anatomical variations, postoperative care, and soft tissue dynamics, key determinants of clinical outcomes, were not modelled in this study. While this investigation provides a standardized biomechanical comparison of fixation techniques, caution is warranted when extrapolating these results to clinical practice. Although finite element analysis has previously been used to investigate Le Fort I fixation systems, future studies combining experimental testing with advanced patient-specific FEA models may further improve our understanding of multidirectional loading patterns and stress distribution across different fixation techniques. This would help optimize plate design and placement. Additionally, long-term fatigue testing under cyclic loading conditions is essential to assess the endurance of plates and screws over time, as repetitive stresses are likely to affect fixation durability and increase the risk of relapse.

Ultimately, clinical trials comparing relapse rates and functional outcomes, such as occlusion and airway dimensions, in patients treated with various fixation techniques, particularly following maxillomandibular advancements, will be crucial to validate the biomechanical findings. It would also be of interest to evaluate Patient-Specific Implants (PSIs), which have emerged as an alternative to conventional off-the-shelf plates. Designed preoperatively based on individual anatomy and surgical planning, PSIs eliminate the need for intraoperative bending and may offer improved accuracy and a better fit [31]. Besides, PSIs were not included in the current study, and future research could benefit from comparing their performance with conventional plates to assess their potential advantages in terms of biomechanical stability, surgical time, and postoperative outcomes.

## Conclusions

This in vitro study demonstrates that hybrid fixation using two Le Fort I plates combined with two L-plates provides the highest biomechanical rigidity in both 5-mm and 10-mm maxillary advancements. Four L-plates also showed high resistance, whereas two-plate constructs were less stable, particularly in larger advancements. These findings suggest that multi-plate fixation may enhance mechanical stability after Le Fort I osteotomy; however, clinical studies are required to determine whether this translates into reduced relapse rates.

## Data Availability

The data presented in this study are available from the corresponding author upon reasonable request.

## Abbreviations

OSA: Obstructive Sleep Apnea
MMA: Maxillomandibular Advancement
BSSO: Bilateral Sagittal Split Osteotomy
PU: Polyurethane
SPSS: Statistical Package for the Social Sciences
SD: Standard Deviation
3D: Three-Dimensional
FEA: Finite Element Analysis
NS: Not significant
